# 2240. Implementation of a Clinical Decision Support Panel for Urine Culture Ordering

**DOI:** 10.1093/ofid/ofac492.1858

**Published:** 2022-12-15

**Authors:** Michael E Yarrington, Fabienne McClellan, Tray Dunkerson, Staci S Reynolds, Christopher R Polage, Becky A Smith, Jessica Seidelman, Sarah S Lewis, Sonali D Advani

**Affiliations:** Duke University Health System, Durham, North Carolina; Duke University Health System, Durham, North Carolina; Duke University Health System, Durham, North Carolina; Duke University School of Nursing, Durham, North Carolina; Duke University School of Medicine, Durham, North Carolina; Duke University, durham, North Carolina; Duke University School of Medicine, Durham, North Carolina; Duke University Medical Center, Durham, North Carolina; Duke University School of Medicine, Durham, North Carolina

## Abstract

**Background:**

Standard urine culture collection techniques have a high risk of false positive results in catheterized patients. IDSA guidelines recommend replacing long-term catheters before specimen collection for cultures. Our goal was to evaluate the impact of clinical decision support (CDS) during the urine culture computerized provider order entry (CPOE) on test use and patient-level outcomes.

**Methods:**

In March 2021, CDS was added to the urine culture CPOE in the electronic health record (EHR). The order panel includes education regarding appropriate clinical indications for urine culturing, and protocolizes urine collection to determine (A) if a urinalysis was ordered, (B) if special patient population (i.e., PICU), (C) the presence and duration of an indwelling urinary catheter, and (D) whether the catheter had difficulty with placement. Depending on the criteria identified in A, B, C, and D, the panel recommends catheter removal prior to urine culture (Figure 1). We retrospectively evaluated the impact of panel implementation in our pre- (7/2020- 3/2021) and post-intervention periods (4/2021- 3/2022) using an interrupted time series analysis with Poisson regression. Outcomes included urine cultures/1000 patient days (pd), number of catheter removal orders, catheter-associated urinary tract infection (CAUTI)/1000 catheter days, and days of antibiotic therapy with urinary tract infection (UTI) indication/1000pd

Order Panel Framework

**Figure:**
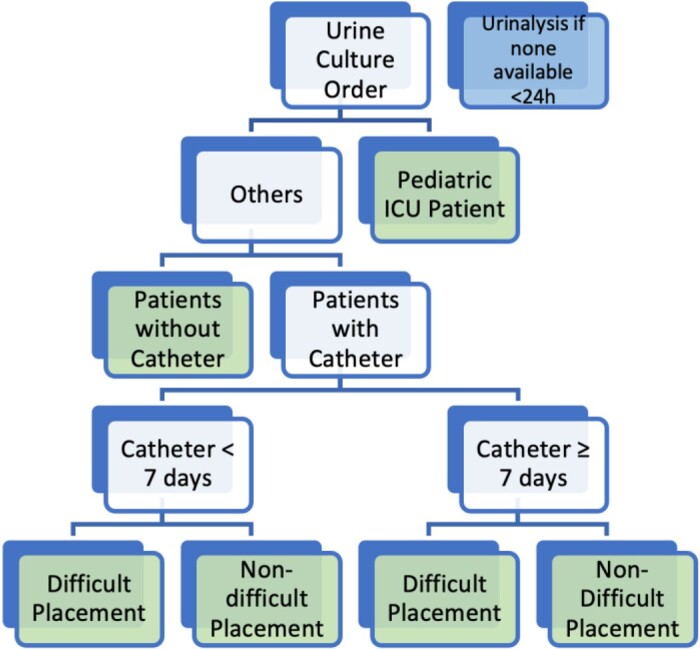

**Results:**

Clinicians selected catheter removal in 183 of 2133 (8.5%) instances of ordering a urine culture when a catheter was detected by the panel (Figure 2). Analysis revealed a significant decrease in urine culture orders (1.1% decrease/month, p< 0.05), a decreasing trend of antibiotic use with UTI indications (Figure 3, 2.8% decrease/month, p< 0.05), but no significant change in CAUTI rates (Figure 4). Evaluation of the safety reporting system revealed no identifiable increase in safety events related to catheter removal or reinsertion.

**Figure:**
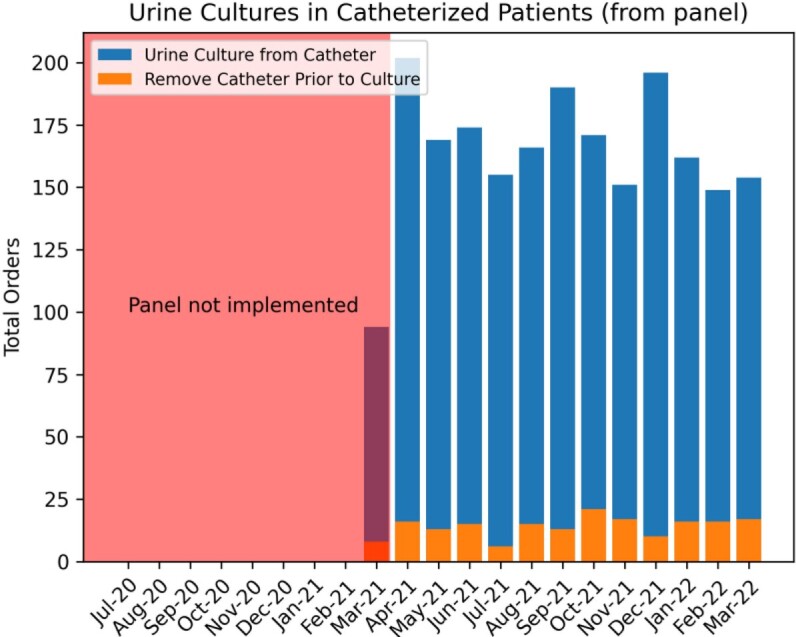

Catheter Associated Urinary Tract Infections

**Figure:**
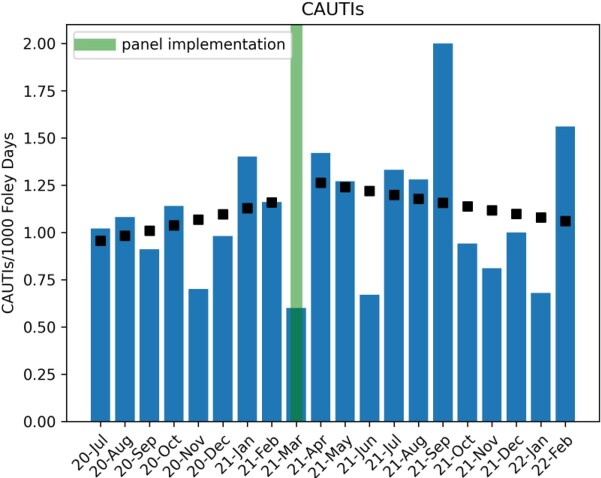

Black boxes indicate Poisson regression model estimates

**Figure:**
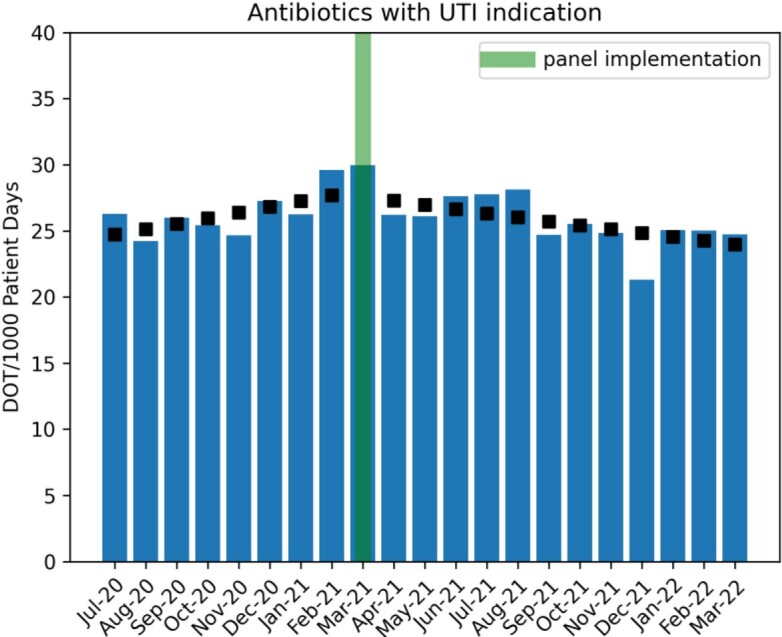

Black boxes indicate Poisson regression model estimates

**Conclusion:**

CDS can aid in improving urine culture practice habits, including removal of long-standing catheters before urine culture. The decreasing trend in total urine culture orders (Figure 5) may reflect a ”culture change” over time to avoid urine cultures in inappropriate instances.

Total Urine Cultures Ordered

**Figure:**
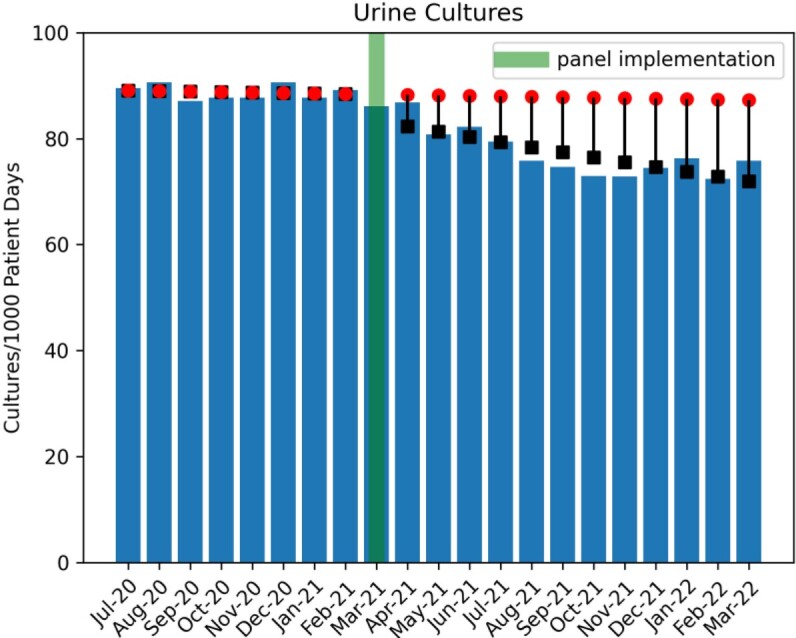

Black boxes indicate Poisson regression model estimates. Red circles indicate predicted outcome without ‘intervention’.

**Disclosures:**

**Sonali D. Advani, MBBS, MPH, FIDSA**, Locus Biosciences: Advisor/Consultant|Locus Biosciences: Honoraria|Sysmex America: Advisor/Consultant.

